# Pen-administered low-dose dasiglucagon vs usual care for prevention and treatment of non-severe hypoglycaemia in people with type 1 diabetes during free-living conditions: a Phase II, randomised, open-label, two-period crossover trial

**DOI:** 10.1007/s00125-023-05909-4

**Published:** 2023-04-11

**Authors:** Christian Laugesen, Ajenthen G. Ranjan, Signe Schmidt, Kirsten Nørgaard

**Affiliations:** 1 Clinical Research, Steno Diabetes Center Copenhagen, Herlev, Denmark; 2 Danish Diabetes Academy, Odense, Denmark; 3 Department of Clinical Medicine, Faculty of Health and Medical Sciences, University of Copenhagen, Copenhagen, Denmark

**Keywords:** Dasiglucagon, Dual-hormone therapy, Hypoglycaemia, Phase 2, Randomised controlled trial, Type 1 diabetes

## Abstract

**Aims/hypothesis:**

Consumption of excess carbohydrates to manage hypoglycaemia can lead to rebound hyperglycaemia and promote weight gain. The objective of this trial was to evaluate the efficacy, safety and feasibility of pen-administered low-dose dasiglucagon for prevention and treatment of non-severe hypoglycaemia in people with type 1 diabetes during free-living conditions.

**Methods:**

Twenty-four adults with insulin pump-treated type 1 diabetes (HbA_1c_ ≤70 mmol/mol [8.5%]) completed a randomised, open-label, two-period crossover study with 2 week periods. During the usual care and dasiglucagon intervention (DASI) periods, participants managed impending and manifested episodes of hypoglycaemia with regular carbohydrate consumption or pen-administered low-dose (80 μg) s.c. dasiglucagon, respectively. Glycaemic control was evaluated using continuous glucose monitoring (Dexcom G6) and event registration of prevention and treatment episodes.

**Results:**

Compared with usual care, the mean difference (95% CI) in the DASI period for time in (3.9–10.0 mmol/l) and below (<3.9 mmol/l) range was 2.4 %-points (−0.7, 5.5) and −0.5 %-points (−1.2, 0.2), respectively. In the DASI period, recovery rate (time from hypoglycaemia treatment to euglycaemia) was 44% (11, 87) faster while total daily carbohydrate intake was reduced by 11% (−18, −3). Dasiglucagon use was safe and well tolerated with mild nausea being the most frequent adverse effect. Among the participants, 96% (*p*<0.0001) were likely to include dasiglucagon in their future routine management of hypoglycaemia.

**Conclusions/interpretation:**

Use of low-dose dasiglucagon to prevent and treat non-severe hypoglycaemia during free-living conditions was safe, fast and efficacious while significantly reducing the total daily carbohydrate intake and yielding high treatment satisfaction.

**Trial registration:**

ClinicalTrials.gov NCT04764968

**Funding:**

The study was an investigator-initiated trial. Zealand Pharma supplied the investigational drug and device and provided financial support for the conduct of the trial.

**Graphical abstract:**

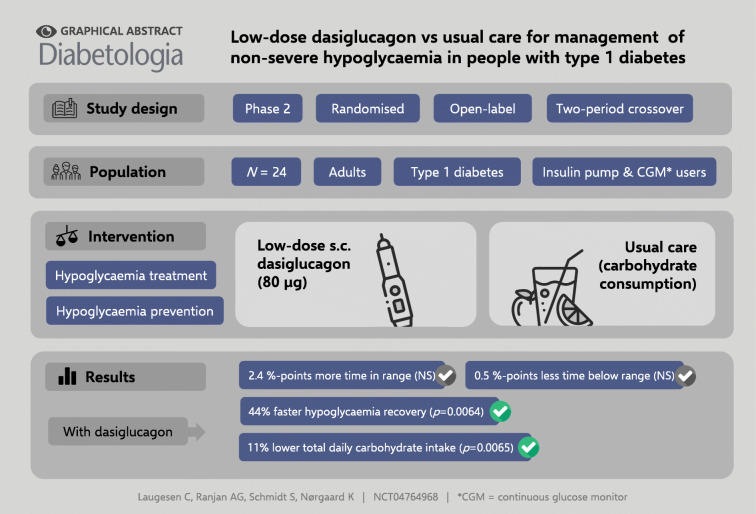

**Supplementary Information:**

The online version contains supplementary material available at 10.1007/s00125-023-05909-4.

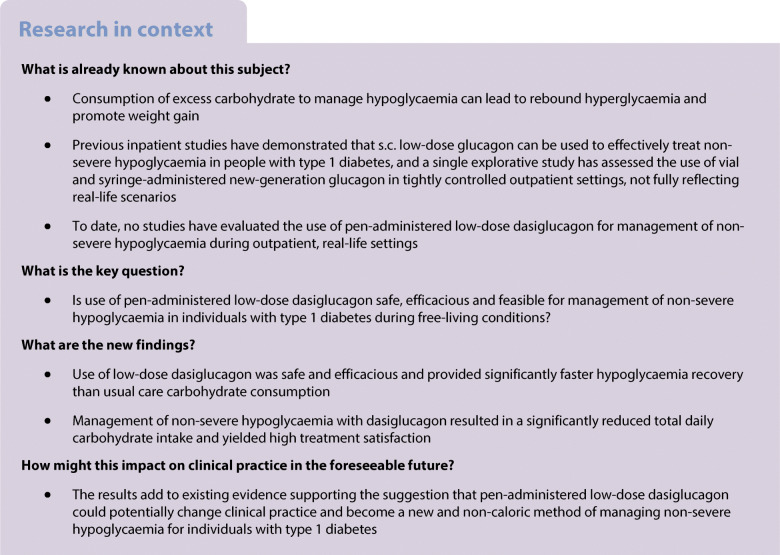



## Introduction

Tight glycaemic control reduces the risk of long-term type 1 diabetes complications but is associated with an increased risk of hypoglycaemia [1, 2]. Although the use of newer insulin analogues, continuous glucose monitors (CGMs) and hybrid closed-loop systems has lowered the risk [3, 4], the majority of individuals with type 1 diabetes still frequently experience non-severe (<3.9 mmol/l [70 mg/dl]) hypoglycaemia [5]. The numerous deleterious physiological and psychological effects of hypoglycaemia are well established [6–8], but reversal of non-severe hypoglycaemia with fast-acting carbohydrates may, in itself, cause undesired health outcomes including hypoglycaemia treatment-induced rebound hyperglycaemia, impaired long-term glycaemic control and weight gain [9–11].

The global obesity pandemic is well established [12], and the consequences for individuals with type 1 diabetes are of particular concern as these increase both diabetes-related and obesity-related complications [13]. Among others, obesity is closely linked to cardiovascular disease, which remains the main cause of mortality in people with type 1 diabetes [14]. Although the mechanisms behind weight gain in people with type 1 diabetes are multifactorial and not fully characterised, defensive snacking to avoid hypoglycaemia and compensatory carbohydrate intake following hypoglycaemia are believed to be key components [13]. Such data emphasise the need for type 1 diabetes treatments not only focusing on glycaemic control, but also addressing the weight management perspective [13].

Studies have previously shown that low doses of s.c. human glucagon can be used as an effective non-caloric alternative to carbohydrates to reverse episodes of non-severe hypoglycaemia [15, 16]. However, as available glucagon preparations until recently required reconstitution before injection, transitioning the concept from research settings into outpatient utilisation and clinical practice has not been possible. Dasiglucagon (Zealand Pharma, Søborg, Denmark), a novel, soluble glucagon analogue, was recently approved by the US Food and Drug Administration for rescue treatment of severe hypoglycaemia (US trade name Zegalogue). Dasiglucagon has a half-life of ~30 min and has, compared with native human glucagon, seven amino acid substitutions ensuring physio-chemical stability and a ready-to-use formulation. Management of non-severe hypoglycaemia using low-dose dasiglucagon has previously been evaluated in inpatient settings [17]. So far, however, no studies have evaluated the use of injection-based low-dose dasiglucagon for management of non-severe hypoglycaemia during outpatient, free-living settings.

The primary objective of this trial was to evaluate the efficacy, safety and feasibility of utilising low-dose s.c. dasiglucagon (administered using an investigational multi-dose reusable pen injector) to treat and prevent episodes of non-severe hypoglycaemia in people with type 1 diabetes during free-living conditions.

## Methods

### Study design

This was an investigator-initiated, single-centre, randomised, open-label, two-period, crossover, investigational drug and device Phase II trial enrolling 24 participants with insulin pump-treated type 1 diabetes. Participants attended a screening visit and three additional study visits scheduled in between and after completing the two 2 week interventional periods (separated by a washout period of 3–7 days).

The study was conducted at the Clinical Research Unit at the Steno Diabetes Center Copenhagen, Herlev, Denmark. It was approved by the Danish Medicines Agency (EudraCT: 2020-005745-16), the Regional Committee on Health Research Ethics (H-21000002) and the Danish Data Protection Agency (P-2021-185) and was monitored by the Good Clinical Practice unit at Bispebjerg and Frederiksberg Hospital, Copenhagen, Denmark. The study was registered at ClinicalTrials.gov (registration no. NCT04764968) and conducted in accordance with the Declaration of Helsinki.

### Participants

Participants were recruited from the outpatient diabetes clinic at Steno Diabetes Center Copenhagen. Key inclusion criteria were age ≥18 years; duration of type 1 diabetes ≥2 years; use of insulin pump therapy (without sensor-augmented insulin suspension/adjustment functionality) for ≥6 months; HbA_1c_ level ≤70 mmol/mol (8.5%); use of real-time or intermittently scanned CGM for ≥3 months and ≥70% during the last 14 days; hypoglycaemic episodes (≤3.9 mmol/l) on ≥4 of the previous 14 days assessed by CGM data; use of carbohydrate counting and the insulin pump bolus calculator; and regular (≥2 times per week) engagement in exercise. Key exclusion criteria included use of glucose-lowering medication other than insulin and hypoglycaemia unawareness (as judged and evaluated by the investigator using the Pedersen-Bjergaard method [18]). A full list of inclusion and exclusion criteria is available in the electronic supplementary material (ESM) Methods.

All individuals provided oral and written informed consent before commencement of any trial-related activity.

### Screening

Screening procedures included routine blood and urine sampling, physical examination, review of medical history and medications, as well as collection of baseline characteristics (age, sex, diabetes duration, duration of insulin pump use, daily insulin dose, height, body weight and blood pressure).

### Randomisation and masking

The order of the two study periods was determined by permuted block randomisation using random blocks of four and six. An allocation table generated by sealedenvelope.com was uploaded to the electronic data capture system, REDCap 12.0.33 (Vanderbilt University) [19, 20], by a person not otherwise involved in the study. Eligible participants were randomly assigned 1:1 to initiate the crossover open-label trial in either the 2 week usual care (UC) period or the 2 week dasiglucagon intervention (DASI) period.

### Procedures

Participants completed two study periods, a 2 week UC period and a 2 week DASI period. Besides the hypoglycaemia management strategy (i.e. UC or low-dose dasiglucagon injection), all trial-related procedures were identical in the two study periods. In both periods, participants wore a Dexcom G6 (Dexcom, San Diego, CA, USA) CGM connected to a Dexcom G6 Receiver. They were worn and used according to applicable guidelines and inserted 24–72 h before the start of each study period. Participants were allowed to use and customise the Dexcom G6 alerts as they preferred; however, the selected settings were required to be used throughout the study, i.e. in both study periods, which participants were informed about prior to initiation of both study periods. To assess the physical activity level, participants also wore a blinded wrist-worn ActiGraph wGT3X-BT (ActiGraph, Pensacola, FL, USA) activity monitor. In addition, participants were instructed to register when they started and ended each exercise session as well as the intensity level (mild, moderate, intense) of these sessions. In both periods, participants were to continue their habitual use of their insulin pump, i.e. entering carbohydrate intake into the pump and, if part of their routine diabetes management, manually suspending or lowering the basal rate whenever needed. Finally, participants were instructed to fill out a questionnaire once a day stating whether they had experienced any episodes of nausea, headache, stomach ache, palpitations, vomiting or other adverse effects. The intensity of adverse effects was scored as mild, moderate or intense.

In the UC period, participants managed episodes of impending or manifested hypoglycaemia as usual by consuming food and/or drinks. Thus, the type, amount and timing of the carbohydrates for hypoglycaemia management were as usual. In all cases where carbohydrates were consumed to prevent or treat an episode of hypoglycaemia (denoted ‘rescue carbohydrates’), participants were instructed to record the exact time and estimated amount of carbohydrates on the Dexcom G6 Receiver. Regular meals and snacks consumed without the intent to prevent or treat an episode of hypoglycaemia were not recorded on the Dexcom G6 Receiver but entered into the insulin pump as usual.

In the DASI period, participants were instructed to manage impending or manifested episodes of hypoglycaemia by administering an s.c. injection of dasiglucagon into a lifted skinfold of the abdominal wall using the injection pen. Unused dasiglucagon cartridges were to be stored refrigerated (2–8°C), whereas cartridges inserted into the pen could be kept at room temperature for up to 1 month (note that Zegalogue [dasiglucagon marketed for treatment of severe hypoglycaemia] can be stored at room temperature for 12 months). The reusable multi-dose pen injector had a minimum and maximum dose of 40 μg and 320 μg, respectively, with incremental doses of 40 μg; however, participants were informed to use a fixed dose of 80 μg in all use scenarios throughout the study period. This dose was selected based on a recent inpatient study of the pharmacodynamic effects of different doses of dasiglucagon [17]. Participants were informed to disinfect the skin and prime the needle (80 μg) before each injection. As each cartridge contained 1 ml of dasiglucagon (4 mg/ml), the number of 80 μg dosings per cartridge totalled 50 (including priming dosings). Participants were allowed to use dasiglucagon in all cases of hypoglycaemia treatment and prevention and, thus, there were no restrictions on the CGM threshold or timing of the dasiglucagon administration. However, participants were instructed to treat any blood glucose level <2.2 mmol/l as usual, i.e. with intake of fast-acting carbohydrates. In cases where the initial dose of dasiglucagon did not restore euglycaemia and/or alleviate the hypoglycaemic symptoms, participants were advised to re-treat with another dose of 80 μg dasiglucagon 15 min after the initial treatment. During the DASI period, participants were allowed to use carbohydrates for treatment and prevention of hypoglycaemia if, for whichever reason, use of dasiglucagon was not feasible or preferable. The exact time and dose of all dasiglucagon administrations were registered on the Dexcom G6 Receiver.

In addition to the initial screening visit and completion of the two study periods, participants attended three in-clinic visits: a midway visit, an end-of-study visit and a follow-up visit scheduled 4 weeks after completion of the DASI period. At the follow-up visit, selected blood samples were collected and analysed for safety purposes. A detailed overview of the different visits and procedures is available in ESM Table 1.

### Outcomes

The primary outcome of the trial was the percentage of CGM-derived time in range (3.9–10.0 mmol/l) during each 2 week period. Secondary glycaemic outcomes included percentage of time below (<3.9 mmol/l) and above (>10.0 mmol/l) range, coefficient of variation and the following event-based outcomes: percentage of cases with successful hypoglycaemia treatment (initial sensor glucose [SG] ≤3.9 mmol/l followed by SG >3.9 mmol/l 30 min post treatment), successful hypoglycaemia treatment without subsequent hyperglycaemia (SG >10 mmol/l within the first 2 h post treatment), successful hypoglycaemia prevention (initial SG >3.9 mmol/l followed by ≤15 consecutive minutes of hypoglycaemia within the first 2 h post treatment), time from hypoglycaemia (<3.9 mmol/l) treatment to euglycaemia (≥3.9 mmol/l) and the incidence rate of supplement carbohydrate administration during the first hour after dasiglucagon administration. Other secondary outcomes included total daily carbohydrate intake, total daily insulin dose and the following participant-reported outcome (PRO): using a four-point Likert scale (very likely, likely, unlikely, very unlikely), participants answered the question, ‘How likely is it that you, given the option, would include dasiglucagon as part of your routine hypoglycaemia management?’ (with ‘very likely’ and ‘likely’ being defined as favourable outcomes). Adverse event outcomes included number of questionnaire-derived adverse events (nausea, headache, stomach ache, palpitations, injection site pain), investigational device failures/malfunctions and serious adverse events.

### Statistical analysis

As this was an exploratory study, the sample size was selected for feasibility and, thus, was not powered to demonstrate a statistically significant difference in the primary outcome. Baseline characteristics are summarised using means (with SD) or proportions. Assessments of period-level continuous outcomes (e.g. time in range, insulin dose, carbohydrate intake etc.) are presented as means (SD) and mean difference, whereas event-based (e.g. cases with successful hypoglycaemia treatment, time from treatment to euglycaemia etc.) outcomes are presented as either rate ratio (time-to-event outcomes) or OR (dichotomous outcomes). All outcomes are presented as adjusted results together with their 95% CI. For continuous endpoints, the treatment effect was evaluated by comparing treatment groups using a linear mixed model that included the factors treatment (two levels), sequence (two levels), period (two levels) and a random participant effect. Where applicable, outcomes were logarithmically transformed to achieve a normal distribution. In cases where the outcome distribution remained skewed, a Wilcoxon signed-rank test was used. Event-based outcomes were evaluated using either a proportional hazards regression model with γ-distributed random effect (time-to-event outcomes) or a logistic regression model with random participant effect (dichotomous outcomes), both models using sequence, period and event baseline SG value as a covariate. The PRO was analysed using the exact binomial test. Statistical analyses were performed in SAS 9.4 (SAS Institute, Cary, NC, USA). A two-sided *p* value of 0.05 was considered statistically significant.

## Results

Between 19 May 2021 and 18 Nov 2021, 26 participants were screened for eligibility, of whom 24 were enrolled and randomly assigned to initiate the crossover trial in either the DASI period (*n*=11) or the UC period (*n*=13). All participants completed the trial and were included in the safety and efficacy analysis (ESM Fig. 1).

Baseline characteristics are shown in Table 1. Participants (58% women) had a mean age of 47 (SD 15) years, duration of type 1 diabetes of 27 (SD 13) years, BMI of 28.3 (SD 4.2) kg/m^2^, total daily insulin dose of 45 (SD 20) U and HbA_1c_ level of 56 (SD 6) mmol/mol (7.3% [SD 0.5]).

**Table 1:** Baseline characteristics

Characteristic	
Age, years	47 ± 15
Sex	
Male	10 (42)
Female	14 (58)
Duration of diabetes, years	27 ± 13
Weight, kg	84.2 ± 14.2
BMI, kg/m^2^	28.3 ± 4.2
Systolic BP, mmHg	130 ± 15
Diastolic BP, mmHg	82 ± 7
HbA_1c_, mmol/mol	56 ± 6
HbA_1c_, %	7.3 ± 0.5
Duration of insulin pump use, years	8 ± 1
Total daily insulin dose, U	45 ± 20
Basal, U	21 ± 10
Bolus, U	24 ± 11
Insulin pump use	
Omnipod	14 (58)
Medtronic	5 (21)
Tandem	4 (17)
Other	1 (4)
Sensor use^a^	
FreeStyle Libre	23 (96)
Dexcom G6	1 (4)
Insulin	
Insulin aspart	21 (88)
Faster insulin aspart	3 (13)

Data are mean ± SD or *n* (%)

Percentages have been rounded and might therefore not add up to 100%

^a^Sensor use before trial enrolment

ESM Fig. 2 illustrates the use of dasiglucagon and rescue carbohydrates in the two study periods and ESM Table 2 provides a further subdivision of the interventions. In the 2 week DASI period, participants administered dasiglucagon a total of 320 times (median per participant=11), whereas rescue carbohydrates were consumed a total of 104 times (median per participant=2). For 91% of the dasiglucagon administrations, the advised dose of 80 μg was used; in the remaining 9% of the cases, participants used either a lower or higher dose. Dasiglucagon was used roughly equally to treat (48%) and prevent (52%) hypoglycaemia. In 243 (76%) cases, participants administered dasiglucagon a single time to treat or prevent the hypoglycaemic event; the remaining 77 (24%) dasiglucagon injections were part of a cluster of administrations (≤30 min to an adjacent administration). In the UC period, participants consumed rescue carbohydrates a total of 498 times (median per participant=16). The splits in percentage of cases between intervention type (treatment vs prevention) and number of administrations (single vs cluster) were similar to the DASI period (ESM Fig. 2).

The overall physical activity level of the participants, as measured by the daily energy expenditure and metabolic equivalents (METs), were not different between the two study periods (ESM Table 3). During the trial, participants performed exercise a total of 262 times (median per participant=8) in the DASI period and 268 times (median per participant=7) in the UC period. The intensity level of the exercise sessions (mild, moderate, intense) was similar between study periods.

### Glycaemic and non-glycaemic outcomes

**Fig. 1:**
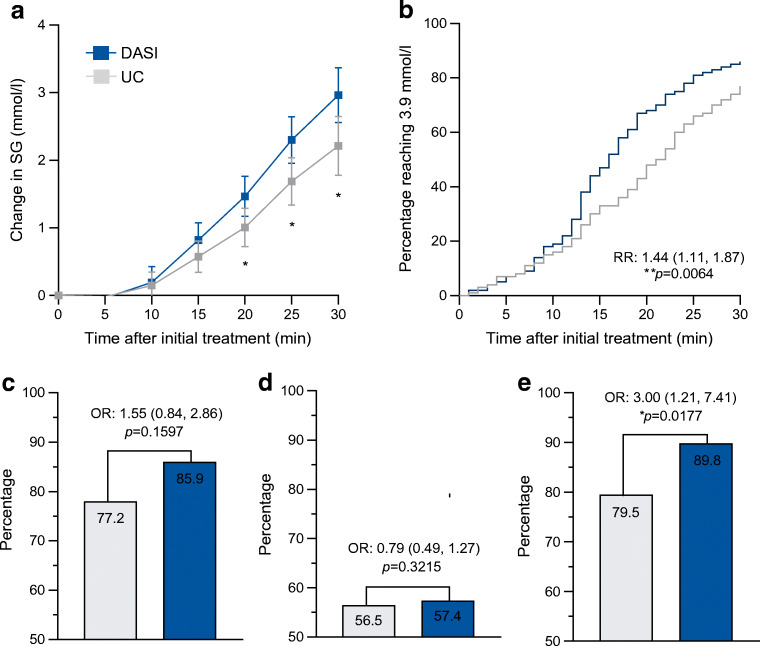
Efficacy profile. Blue lines/bars represent the DASI period; grey lines/bars represent the UC period. (**a**) Mean (±95% CI) change in SG after initial hypoglycaemia treatment. (**b**) One minus Kaplan–Meier plot of time from initial hypoglycaemia treatment (SG <3.9 mmol/l) to reaching euglycaemia (SG ≥3.9 mmol/l). Difference between the two periods is shown as RR. (**c**) Percentage of successful cases of hypoglycaemia treatment (initial SG ≤3.9 mmol/l followed by SG >3.9 mmol/l 30 min post treatment). (**d**) Percentage of successful cases of hypoglycaemia treatment without subsequent hyperglycaemia (SG >10 mmol/l within the first 2 h post treatment). (**e**) Percentage of successful cases of hypoglycaemia prevention (initial SG >3.9 mmol/l followed by ≤15 consecutive minutes of hypoglycaemia within the first 2 h post treatment). Statistical notation used: *p<0.05; **p<0.01. RR, rate ratio

Glycaemic and non-glycaemic outcomes are shown in Table 2 and event-based outcomes are illustrated in Fig. 1. Compared with the UC period, participants spent 2.4 %-points (95% CI −0.7, 5.5) more time in the target range in the DASI period (UC: 61.1%, DASI: 63.5%, *p*=0.1286). Similarly, participants spent 0.5 %-points (95% CI −1.2, 0.2) less time below range (UC: 3.1%, DASI: 2.5%, *p*=0.1622) and 1.9 %-points (95% CI −5.4, 1.6) less time above range (UC: 35.8%, DASI: 33.9%, *p*=0.2772).

**Table 2:** Glycaemic and non-glycaemic endpoints

Endpoint	UC period	DASI period	Measure of comparison (95% CI)	*p*
Time in range (3.9–10.0 mmol/l), %	61.1 ± 15	63.5 ± 11	MD 2.4 (−0.7, 5.5)	0.1286
Time below range (<3.9 mmol/l), %	3.1 ± 4	2.5 ± 3	MD −0.5 (−1.2, 0.2)	0.1622
Time above range (>10.0 mmol/l), %	35.8 ± 17	33.9 ± 13	MD −1.9 (−5.4, 1.6)	0.2772
Coefficient of variation, %	35.2 ± 5	35.6 ± 4	MD 0.4 (−1.0, 1.8)	0.5503
Successful cases of hypoglycaemia treatment^a^, *n*/*N* (%)	173/224 (77)	146/170 (86)	OR 1.55 (0.84, 2.86)	0.1597
Successful cases of hypoglycaemia treatment without subsequent hyperglycaemia^b^, *n*/*N* (%)	126/223 (57)	97/169 (57)	OR 0.79 (0.49, 1.27)	0.3215
Successful cases of hypoglycaemia prevention^c^, *n*/*N* (%)	132/166 (80)	149/166 (90)	OR 3.00 (1.21, 7.41)	0.0177
Recovery rate (median [IQR] time from treatment to euglycaemia^d^), min	21 [13–30]	16 [12–23]	RR 1.44 (1.11, 1.87)	0.0064
Total daily carbohydrate intake, g	191 ± 66	171 ± 59	MD −20 (−34, −6)	0.0065
Daily rescue carbohydrate intake^e^, g	28 ± 22	6 ± 9	MD −22 (−29, −14)	<0.0001
Incidence rate of carbohydrate intake 1 h after dasiglucagon administration, *n*/*N* (%)	NA	22/282 (8)	NA	NA
Total daily insulin dose, U	46.4 ± 20	47.1 ± 22	MD 0.7 (−1.5, 2.9)	0.5148

Data are mean ± SD or *n/N* (%) unless stated otherwise

Analyses are adjusted for sequence, period and baseline SG value (event-based endpoints only)

^a^Defined as initial SG ≤3.9 mmol/l followed by SG >3.9 mmol/l 30 min post treatment

^b^Defined as successful hypoglycaemia treatment without subsequent hyperglycaemia (SG >10 mmol/l within the first 2 h post treatment)

^c^Defined as initial SG >3.9 mmol/l followed by ≤15 consecutive minutes of hypoglycaemia within the first 2 h post treatment

^d^Defined as time from initial hypoglycaemia (<3.9 mmol/l) treatment to euglycaemia (≥3.9 mmol/l)

^e^Defined as carbohydrates consumed to treat or prevent a hypoglycaemic event

MD, mean difference; NA, not applicable; RR, rate ratio

When evaluating the event-based outcomes, the recovery rate (time from hypoglycaemia treatment to euglycaemia) was 44% (95% CI 11, 87; *p*=0.0064) faster in the DASI period (16 min [IQR 12, 23]) compared with the UC period (21 min [IQR 13, 30]). Similarly, the percentage of successful cases of hypoglycaemia treatment was numerically higher in the DASI period (UC: 77%, DASI: 86%, OR 1.55 [95% CI 0.84, 2.86], *p*=0.1597), whereas successful hypoglycaemia treatment without subsequent hyperglycaemia did not differ between study periods (UC: 57%, DASI: 57%, OR 0.79 [95% CI 0.49, 1.27], *p*=0.3215). Finally, the percentage of successful cases of hypoglycaemia prevention was also higher in the DASI period (UC: 80%, DASI: 90%, OR 3.00 [95% CI 1.21, 7.41], *p*=0.0177).

During the DASI period, the total daily carbohydrate intake was significantly reduced by 20 g (95% CI −34, −6; *p*=0.0065), corresponding to 11% (95% CI −18, −3). The difference was caused by a significantly lower intake of daily rescue carbohydrates (−22 g [95% CI −29, −14], *p*<0.0001), whereas there was no difference in the amount of consumed meal carbohydrates.

### Adverse events

Adverse events were reported by 42% (number of events=56) of the participants in the UC period and 46% (number of events=90) in the DASI period (Table 3). In the DASI period, nausea and headache were the most frequently reported adverse events, and there were no cases of vomiting, injection site pain or severe hypoglycaemic episodes. The intensity level of the adverse events was predominantly (81%) mild in the DASI period, and the numbers of events classified as moderate and intense were not higher than in the UC period. Blood samples collected at the follow-up safety visit showed no changes from baseline in electrolytes, liver enzymes and renal function (ESM Table 4). In general, the side effects reported for dasiglucagon were consistent with the known profile for low-dose glucagon use, and no serious, unexpected or device-related adverse events were observed throughout the trial.

**Table 3:** Adverse events for each 2 week interventional period

	UC period			DASI period		
	Participants, *n*	Participants, %	Events, *n*	Participants, *n*	Participants, %	Events, *n*
All AEs	10	42	56	11	46	90
SAEs	0	0	0	0	0	0
Type						
Nausea	4	17	17	8	33	34
Vomiting	1	4	1	0	0	0
Headache	8	33	22	10	42	36
Stomach ache	3	13	15	4	17	16
Palpitations	1	4	1	3	13	4
Injection site pain	NA	NA	NA	0	0	0
Device malfunction	NA	NA	NA	0	0	0
Intensity of AEs^a^						
Mild (%^b^)	NA	NA	30 (55)	NA	NA	71 (81)
Moderate (%^b^)	NA	NA	22 (40)	NA	NA	13 (15)
Intense (%^b^)	NA	NA	3 (5)	NA	NA	4 (5)

^a^The intensity levels of three AEs (one in the UC period, two in the DASI period) were not specified by the participants

^b^Proportion of all AEs

AE, adverse event; NA, not applicable; SAE, serious adverse event

### Participant-reported outcome

The PRO demonstrated that after completing the study, the vast majority of participants would use low-dose dasiglucagon in their future daily diabetes management if available; 96% responded that they were either very likely (88%) or likely (8%) to use dasiglucagon as part of their routine hypoglycaemia management, whereas 4% (one person) and none answered that this was unlikely (due to nausea) or very unlikely, respectively (*p*<0.0001).

## Discussion

This investigational drug and device trial demonstrated that low-dose s.c. dasiglucagon was a safe, effective and feasible alternative to usual carbohydrate consumption for treatment and prevention of non-severe hypoglycaemia during free-living conditions. Utilisation of dasiglucagon for hypoglycaemia management resulted in faster hypoglycaemia recovery; higher hypoglycaemia treatment and prevention success; markedly reduced carbohydrate intake; high treatment satisfaction; and modest, non-significant improvements in percentage of time in, below and above range. The side effects profile of dasiglucagon was in line with those previously reported for low-dose glucagon, with mild and transient nausea being the most frequent adverse event.

Use of low-dose glucagon to manage hypoglycaemia has a number of potential merits. As hypoglycaemia adversely impacts mental health and well-being [6–8], rapid restoration of euglycaemia and relief of hypoglycaemic symptoms are important [21]. The fast hypoglycaemia recovery rate with dasiglucagon shown in the study supports the findings from a recent inpatient Phase II study comparing the pharmacodynamic profiles of two doses of s.c. dasiglucagon (80 and 120 μg) with 15 g of oral glucose for hypoglycaemia management [17]. The study demonstrated that both doses of dasiglucagon had a significantly faster glucose-elevating profile than oral glucose while producing a peak glucose level very similar to the oral glucose intervention. Therefore, there is now evidence from both controlled inpatient settings and outpatient free-living conditions that use of low-dose dasiglucagon can produce a faster hypoglycaemia recovery than consumption of fast-acting carbohydrates.

In this study, the occurrence of rebound hyperglycaemia following successful hypoglycaemia treatment was not lower in the DASI period compared with UC. At the same time, restoration of euglycaemia within 30 min after initial treatment, although more effective than in the UC period, was not met in all cases in the DASI period. The findings prompt a discussion of utilising a flexible dosing regimen of dasiglucagon. In the current trial, the instructed dose of 80 μg was effective and well-suited for many participants and hypoglycaemic episodes. However, in some cases, data suggest that either a higher or lower dose would have been favourable. As is also evident from hypoglycaemia management in clinical practice, inter- and intraindividual differences in carbohydrate requirements exist depending on initial glucose concentration, preceding physical activity, insulin on board and other variables. Therefore, a flexible dosing regimen for dasiglucagon, allowing individuals to tailor the dose for each use case, could potentially further optimise the glycaemic control by ensuring sufficient efficacy while minimising the risk of subsequent hyperglycaemia.

Due to the growing prevalence of obesity in individuals with type 1 diabetes and the fact that hypoglycaemia-associated carbohydrate consumption may play an important role, there has been increasing focus on developing treatment strategies focusing not only on glycaemic control, but also on weight management [11, 13]. In this trial, the total daily carbohydrate intake was reduced by 11% in the DASI period, a difference caused by a significant lowering of the rescue carbohydrate consumption. The duration of this trial did not allow for assessment of changes in body weight; this important endpoint needs to be evaluated in long-term studies.

The present trial provides a number of new insights. It is the first study to evaluate the use of pen-administered low-dose dasiglucagon for management of non-severe hypoglycaemia in outpatient settings. Previously, Haymond and colleagues conducted an exploratory outpatient study investigating the use of syringe-administered glucagon (Gvoke, Xeris Pharmaceuticals, Chicago, IL, USA) for management of non-severe hypoglycaemia [22]. More recently, Algeffari and colleagues evaluated the use of syringe-administered, reconstituted powder-formulation low-dose GlucaGen (Novo Nordisk, Søborg, Denmark) in fasted individuals with type 1 diabetes during Ramadan [23]. Both studies provided valuable insights into the efficacy and feasibility of managing non-severe hypoglycaemia using low doses of glucagon in outpatient settings. A number of important differences, however, distinguish the two studies from this present study. Whereas the present study used an investigational multi-dose reusable pen, the previous studies both used vial and syringe to administer glucagon which, although feasible from a proof-of-concept point view, does not reflect the expected real-world utilisation of the treatment concept. Second, both studies used a strict protocol for the timing, composition and amount of the carbohydrates in the control arm. Although the approach indeed has some strengths, it does not, as stated by the authors, fully reflect how individuals usually treat hypoglycaemic events during real-life conditions. In this trial, participants managed hypoglycaemia in the UC period as they routinely do, creating a representative frame of reference which is decisive in the effort to evaluate the comparable efficacy of the dasiglucagon intervention, both on a period level and on the event level. Another strength of this trial is the extensive amount of event data available as a result of the frequent use of dasiglucagon (6.7 glucagon administrations per participant per week compared with 1.3–1.4 in previous trials), enabling a more robust evaluation of the efficacy profile. Despite the frequent use of dasiglucagon, however, participants did use carbohydrates to manage hypoglycaemia in some cases in the DASI period (25% of the cases equalling 6 g/day). This is expected and clearly illustrates that for some participants, use of carbohydrates may still be preferred in some situations. Finally, the present trial is the first to evaluate the effect of the glucagon intervention on the total carbohydrate intake. The study demonstrated a significant and clinically meaningful reduction in the total carbohydrate intake, the first step in validating one of the key believed merits of the treatment concept.

The study has some limitations. As all participants used insulin pumps (selected to ensure precise insulin administration data) and stand-alone CGMs, were moderately physically active and had regular episodes of non-severe hypoglycaemia, additional studies are required to generalise the findings to the broader population with type 1 diabetes, especially to individuals using other treatment modalities. Another limitation is the study duration. Although the two 2 week intervention periods were sufficient to generate important and clinically meaningful findings, endpoint evaluation in longer trials is required. Additionally, a longer trial duration would have allowed participants to better familiarise themselves with the utilisation and treatment response of dasiglucagon which could potentially improve glycaemic control further. The current sample size was selected for feasibility and was not powered to demonstrate a significant difference in the primary outcome. However, the present trial was able to demonstrate statistically significant differences in a range of outcomes that have substantial clinical implications. Nevertheless, larger studies are required to generate more robust data.

Looking ahead, more questions need to be addressed. Larger studies of longer duration are required to assess the long-term efficacy and safety, changes in body weight and a more extensive set of PROs. In addition, utilisation of a flexible dosing regimen and inclusion of individuals using other treatment modalities, including both advanced hybrid closed-loop users and multiple daily injection users, are required to address and reflect the full potential of using low-dose dasiglucagon to manage hypoglycaemia.

In conclusion, this study demonstrated that use of pen-administered low-dose dasiglucagon, a novel, ready-to-use glucagon analogue, to treat and prevent non-severe hypoglycaemia during free-living conditions was safe, fast and efficacious while significantly reducing the total daily carbohydrate intake and yielding high treatment satisfaction. The findings support the continued study of utilising low-dose glucagon as a non-caloric alternative to carbohydrates for management of hypoglycaemia in individuals with type 1 diabetes.

## Supplementary information

ESM 1 (PDF 261 kb)

## Data Availability

The dataset generated from this study will not be made publicly available because of data protection regulations, but investigators (whose proposed use of the data has been approved by an independent review committee identified for this purpose) can request access to de-identified participant data or anonymised clinical study reports 24 months after publication by contacting the corresponding author. The trial investigators will review the request and, if accepted, data will be shared upon approval from relevant bodies in the Capital Region of Denmark governing sharing of trial data. The study protocol will be made available on ClinicalTrials.gov after the publication of this study.

## Abbreviations

CGM: Continuous glucose monitor
DASI: Dasiglucagon intervention
SG: Sensor glucose
UC: Usual care
PRO: Participant-reported outcome
