# Supplementary material for: Pen-administered low-dose dasiglucagon vs usual care for prevention and treatment of non-severe hypoglycaemia in people with type 1 diabetes during free-living conditions: a Phase II, randomised, open-label, two-period crossover trial
Source: Diabetologia. 2023 Apr 11;66(7):1208–17. doi: 10.1007/s00125-023-05909-4 (PMC10244275; doi:10.1007/s00125-023-05909-4)
Supplement: Supplementary file 1 — (PDF 261 kb) [file 125_2023_5909_MOESM1_ESM.pdf]

Electronic Supplementary Material (ESM)

Pen-administered low-dose dasiglucagon vs usual care for prevention and treatment of non-severe hypoglycaemia in people with type 1 diabetes during free-living conditions: a phase 2, randomised, open-label, two-period crossover trial

Table of Contents

ESM Methods....2

ESM Table 1. Study visit procedures....3

ESM Table 2. Use of dasiglucagon and rescue carbohydrates....4

ESM Table 3. Activity monitor data and exercise sessions....5

ESM Table 4. Safety blood samples....6

ESM Fig 1. Trial profile....7

ESM Fig 2. Use of dasiglucagon and rescue carbohydrates....8

ESM Methods

Inclusion criteria:

- Age ≥ 18 years
- Type 1 diabetes ≥ 2 years
- Use of insulin pump therapy (without sensor-augmented insulin suspension/adjustment functionality) ≥ 6 months
- Use of CGM (real-time or intermittently scanned) ≥ 3 months and ≥ 70% during the previous 14 days
- HbA_{1c} ≤ 8.5% (70 mmol/mol)
- Performs aerobic exercise ≥ 2 times per week (self-reported) and desires to exercise per American Diabetes Association guidelines (150 minutes per week) during the study.
- Use of carbohydrate counting and bolus calculator (self-reported)
- Sensor glucose level < 3.9 mmol/l on ≥ 4/14 previous days assessed by CGM data

Exclusion criteria:

- Use of anti-diabetic medicine (other than insulin), corticosteroids or other drugs affecting glucose metabolism during the study period and within 30 days prior to study start
- Known or suspected allergies to glucagon or related products
- History of hypersensitivity or allergic reaction to dasiglucagon or any of the excipients
- Patients with pheochromocytoma or insulinoma
- Hypoglycemia unawareness
- Females of childbearing potential who are pregnant, breast-feeding or intend to become pregnant or are not using adequate contraceptive methods
- Inability to understand the individual information and to give informed consent
- Current participation in another clinical trial that, in the judgment of the investigator, will compromise the results of the study or the safety of the subject
- Concomitant medical or psychological conditions identified through review of medical history, physical examination and clinical laboratory analysis that, according to the investigator's assessment, makes the individual unsuitable for study participation

ESM Table 1. Study visit procedures

Week #	-2	1	2	3	4	5	6	10[a]
Days ±	14	-	-	3	-	-	7	7
	Screening	Study period 1		Midway visit	Study period 2		End-of-study visit	Follow-up visit
Blood samples	X							X
Urine samples	X							
ECG	X							
Weight, height, blood pressure	X							
Download of CGM data				X			X	
Download of insulin pump data				X			X	
Download of ActiGraph data				X			X	
AE questionnaire collection				X			X	
Patient-reported outcome questionnaire							X	

ECG=electrocardiogram. CGM=continuous glucose monitor. AE=adverse event. [a]Follow-up visit was scheduled 4 weeks after the end of the dasiglucagon period.

ESM Table 2. Use of dasiglucagon and rescue carbohydrates

	UC period	DASI period	
Intervention	CHO	DASI	CHO
Total treatments[a]	271 (100%)	149 (100%)	42 (100%)
Single treatment	191 (70%)	130 (87%)	25 (60%)
Clustered treatments[b]	80 (30%)	19 (13%)	17 (40%)
First in cluster	33 (41%)	12 (63%)	3 (18%)
Repeat in cluster	47 (59%)	7 (37%)	14 (82%)
Initial treatment[c]	224	142	28
Total preventions[d]	221 (100%)	164 (100%)	60 (100%)
Single prevention	175 (80%)	109 (66%)	39 (65%)
Clustered prevention†	46 (21%)	55 (34%)	21 (35%)
First in cluster	27 (59%)	25 (45%)	8 (38%)
Repeat in cluster	19 (41%)	30 (55%)	13 (62%)
Initial prevention‡	202	134	47

Data are n (%). UC=usual care. DASI=dasiglucagon. CHO=carbohydrates consumed to prevent or treat an episode of hypoglycaemia. [a]Interventions administered at a SG level ≤3·9

mmol/l. [b]Interventions administered ≤30 min to an adjacent intervention. [c]Sum of single interventions and first intervention in a cluster. [d]Interventions administered at a sensor glucose level >3·9 mmol/l.

ESM Table 3. Activity monitor data and exercise sessions

	UC period	DASI period
Energy expenditure*, kcal/day	1849 (150)	1943 (117)
METs/day*	1·52 (0·04)	1·53 (0·03)
Total exercise sessions†, n (median per subject)	268 (7)	262 (8)
Light, n (%)	112 (42%)	125 (48%)
Moderate, n (%)	83 (31%)	72 (27%)
Intense, n (%)	70 (26%)	64 (24%)
Unclassified, n (%)	3 (1%)	1 (0%)

Data are mean (SE) or n (%) unless stated otherwise. UC=usual care. DASI=dasiglucagon. METs=metabolic equivalents. *Measured by a wrist-worn activity monitor (ActiGraph wGT3X-BT). †Manually registered by the participants.

ESM Table 4. Safety blood samples

	4-week follow-up[a]	Change from baseline
eGFR, mL/min/1,73 m^2	93 (3)	0 (2)
Creatinine, µmol/L	72 (3)	1 (1)
Potassium, mmol/l	4·0 (0·1)	0·0 (0·1)
Calcium, mmol/l	2·3 (0·0)	0·0 (0·0)
ALAT, U/L	23 (2)	0 (2)
ASAT, U/L	25 (1)	1 (2)

Data are mean (SE). [a]Follow-up blood samples collected 4 weeks after the end of the dasiglucagon period.

ESM Fig 1. Trial profile

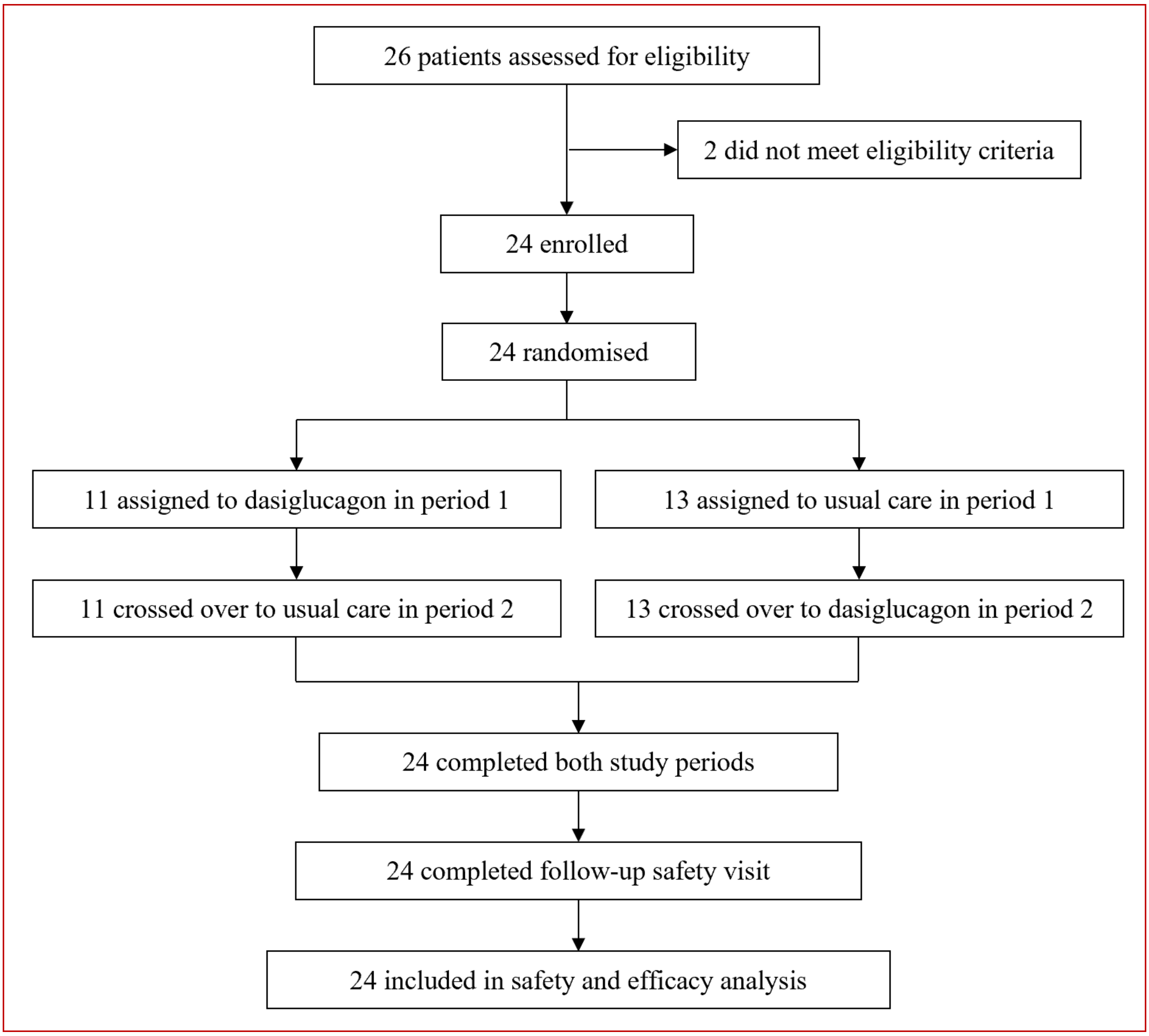

ESM Fig 2. Use of dasiglucagon and rescue carbohydrates

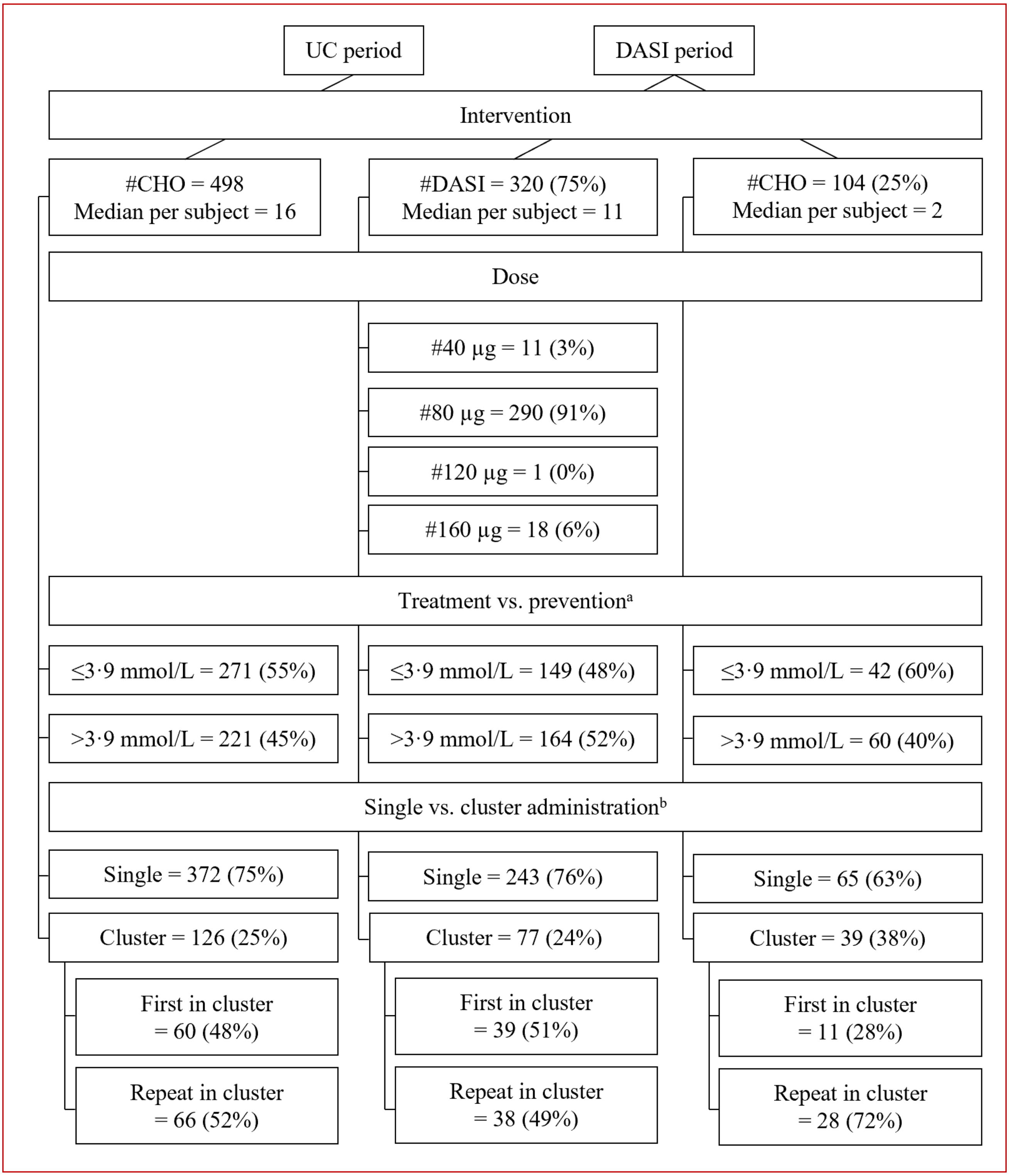

Data are n (%). UC=usual care. DASI=dasiglucagon. CHO=carbohydrates consumed to prevent or treat an episode of hypoglycaemia. [a]Treatment and prevention events were defined as interventions administered at a sensor glucose level ≤3·9 mmol/l or >3·9 mmol/l,

respectively. 15 interventions (6 in UC, 9 in DASI) were not categorised as treatment/prevention as they had no previous CGM value within the previous 15 min.

[b]Interventions administered ≤30 min to an adjacent intervention was defined as being a part of a cluster of interventions. First in cluster was defined as the first intervention in a cluster of interventions. Repeat in cluster was defined as an intervention in a cluster that was not the first intervention. Supplementary table 2 contains a detailed subdivision of the treatment and prevention interventions.
